# Genomic degradation of a young Y chromosome in *Drosophila miranda*

**DOI:** 10.1186/gb-2008-9-2-r30

**Published:** 2008-02-12

**Authors:** Doris Bachtrog, Emily Hom, Karen M Wong, Xulio Maside, Pieter de Jong

**Affiliations:** 1 Division of Biological Sciences, University of California San Diego, La Jolla, CA 92093, USA; 2 Institute of Evolutionary Biology, School of Biological Sciences, University of Edinburgh, Edinburgh EH9 3JT, UK; 3 Children's Hospital Oakland Research Institute, Oakland, CA 94609, USA; 4 Current address: Grupo de Medicina Xenómica, Instituto de Medicina Legal, CIBERER, Universidade de Santiago de Compostela, 15782 Santiago de Compostela, Spain

## Abstract

**Background:**

Y chromosomes are derived from ordinary autosomes and degenerate because of a lack of recombination. Well-studied Y chromosomes only have few of their original genes left and contain little information about their evolutionary origin. Here, we take advantage of the recently formed neo-Y chromosome of *Drosophila miranda *to study the processes involved in Y degeneration on a genomic scale.

**Results:**

We obtained sequence information from 14 homologous bacterial artificial chromosome (BAC) clones from the neo-X and neo-Y chromosome of *D. miranda*, encompassing over 2.5 Mb of neo-sex-linked DNA. A large fraction of neo-Y DNA is composed of repetitive and transposable-element-derived DNA (20% of total DNA) relative to their homologous neo-X linked regions (1%). The overlapping regions of the neo-sex linked BAC clones contain 118 gene pairs, half of which are pseudogenized on the neo-Y. Pseudogenes evolve significantly faster on the neo-Y than functional genes, and both functional and non-functional genes show higher rates of protein evolution on the neo-Y relative to their neo-X homologs. No heterogeneity in levels of degeneration was detected among the regions investigated. Functional genes on the neo-Y are under stronger evolutionary constraint on the neo-X, but genes were found to degenerate randomly on the neo-Y with regards to their function or sex-biased expression patterns.

**Conclusion:**

Patterns of genome evolution in *D. miranda *demonstrate that degeneration of a recently formed Y chromosome can proceed very rapidly, by both an accumulation of repetitive DNA and degeneration of protein-coding genes. Our data support a random model of Y inactivation, with little heterogeneity in degeneration among genomic regions, or between functional classes of genes or genes with sex-biased expression patterns.

## Abstract 2

Study of the recently formed neo-Y chromosome of Drosophila miranda demonstrate that degeneration of a recently formed Y-chromosome can proceed very rapidly.

## Background

In many animal and plant species, sex is determined by a pair of morphologically and genetically distinct sex chromosomes (X and Y chromosomes in species with male heterogamety [1-3]). A striking common feature of many taxa is the almost complete erosion of genes from the Y chromosome (or W chromosome, with female heterogamety). The Y chromosome also often contains an unusual abundance of repetitive DNA sequences [1,4]. Sex chromosomes evolved independently many times from an ordinary pair of autosomes that stopped recombining with each other after acquiring a sex-determining role [2,3]. In the absence of recombination, these originally homologous chromosomes continued to differentiate. The X chromosome, which continues to recombine in females, maintains most of its ancestral genes whereas the non-recombining region of the Y chromosome degenerates [2,3]. Various population genetic processes can lead to the observed loss of gene function from a non-recombining chromosome [1-3].

For example, the recently completed sequence of the human Y chromosome [5] has revealed that the 24 Mb male-specific portion of this chromosome contains only 78 protein-coding genes. However, old Y chromosomes, such as the human Y (about 300 million years old), retain few traces of the evolutionary processes that led to their degeneration. The best hope for identifying the causes of Y chromosome evolution is to investigate a young Y chromosome that is still in the process of degeneration.

*Drosophila miranda*, a close relative of *D. pseudoobscura*, has a recently formed new sex chromosome pair (called the neo-sex chromosomes). Due to a fusion of an autosome to the Y chromosome about one million years ago [6], a neo-Y chromosome has been formed. Its unfused homolog, which is in one copy in males and two copies in females, is called the neo-X chromosome. The neo-Y chromosome is transmitted in association with the ancestral Y chromosome through males only. Since male *Drosophila *lack recombination, this chromosomal arm is completely sheltered from recombination, and thus subject to the same evolutionary forces as a true Y chromosome [7,8]. Interestingly, most of the human Y chromosome is derived from a Y-autosome fusion; thus, the human Y chromosome is also a degenerate neo-Y [9].

Previous studies have shown that the neo-Y chromosome of *D. miranda *is in transition from an ordinary autosome into a degenerate Y chromosome [8,10,11]. About one-third of all genes studied to date are clearly non-functional on the neo-Y, containing frame-shift mutations or stop codons [10]. Most remaining genes show various other signs of degeneration, such as an elevated rate of amino-acid substitutions [6,10-13] and less constraint in regulatory regions [10]. Moreover, *in situ *hybridization experiments and sequence analysis have revealed an accumulation of transposable elements on the neo-Y relative to the neo-X [8,14,15].

However, these previous studies in *D. miranda *have investigated only relatively small genomic regions (mainly 1 kb fragments amplified by PCR and a few clones isolated from a λ genomic library). These studies are biased towards identifying conserved regions between the neo-sex chromosomes, and may greatly underestimate the fraction of non-functional or missing genes on the neo-Y, or the level of intergenic divergence, including the amount of transposable element DNA on the neo-Y. Here, we study the genomic organization of the neo-Y chromosome using sequenced bacterial artificial chromosome (BAC) clones from 7 regions scattered along the neo-sex chromosomes of *D. miranda*, which contain over 100 homologous gene pairs. The BAC sequencing approach here should remove the bias of identifying conserved regions, and allows us to obtain an unbiased estimate on the amount of degeneration of the neo-Y. In addition, this much larger data set also enables us to test which genomic features influence Y degeneration.

In particular, we address the following questions. What fraction of genes is non-functional or missing on the neo-Y? Is selection against amino acid changes reduced on the neo-Y? Are certain classes of genes more prone to degeneration? How abundant are transposable elements on the neo-Y? How much heterogeneity in the amount of degeneration and TE abundance is there among regions? Does an accumulation of TEs trigger degeneration at adjacent protein-coding regions? Are genes with female-biased expression more prone to degeneration on the neo-Y, and male-biased genes more conserved?

## Results and discussion

### Sequence analysis of the evolving sex chromosomes of *D. miranda*

To investigate the genomic organization of the neo-sex chromosomes, a BAC library made from male *D. miranda *was screened. We used a pool of probes for 20 genes that were shown previously to be neo-sex linked [10]. A total of 269 positive BAC clones were identified. BAC clones were end-sequenced using the SP6 and T7 primers. The sequences were blasted against the *D. pseudoobscura *genome sequence to orient the BAC clones along chromosome 3 of *D. pseudoobscura*, which is homologous to the neo-sex chromosomes of *D. miranda*. BAC clones were subsequently typed as being from the neo-X or neo-Y using sex-specific polymorphisms. A total of 202 BAC clones were mapped and oriented to the neo-sex chromosomes of *D. miranda*. Fourteen BAC clones were chosen for sequence analysis, from seven homologous regions on the neo-X and neo-Y that maximized both the number of genes sequenced and the overlap between the neo-X and neo-Y chromosomes (Table 1). Six BAC clones were sequenced using Sanger sequencing technology, and eight BAC clones were sequenced using 454 technology. Assemblies using these two technologies resulted in a very similar number of large contigs containing >500 bp per BAC (that is, roughly 10-20 large contigs for neo-X linked BAC clones, or 30-50 large contigs for neo-Y linked BAC clones; Table 1). The total length in base pairs of large contigs is higher for the neo-X clones than for neo-Y clones (Table 1). This likely reflects the presence of transposable element (TE) fragments and repetitive DNA on the neo-Y chromosome (see below), resulting in poorer sequence assemblies on the neo-Y, and the collapse of homologous repetitive elements into the same contig.

**Table 1:** Genomic regions investigated on the neo-sex chromosomes of *D. miranda*

Probe	Linkage	BAC clone	Position *D. pseudoobscura*	No. of large contigs (>500 bp)	Large contig total length
Cyp6t3*	neo-X	CH229-18P24	2,919,127-3,118,766	61	191,732
	neo-Y	CH229-17G1	2,930,284-2,999,451	48	152,913
					
CG11228^†^	neo-X	CH229-9J14	6,378,633-6,574,929	12	212,381
	neo-Y	CH229-47N8	6,224,922-6,574,929^‡^	40	101,234
					
CG3167^†^	neo-X	CH229-1G8	7,534,774-7,718,283	10	196,418
	neo-Y	CH229-11G24	7,575,595-7,622,144	42	150,425
					
CG2269^†^	neo-X	CH229-38G20	9,562,050-9,772,080	10	215,404
	neo-Y	CH229-20L11	9,560,730-9,624,726	46	136,298
					
RnrS*	neo-X	CH229-21P17	12,046,872-12,252,350	10	219,495
	neo-Y	CH229-3L20	12,166,695-12,235,653	53	166,051
					
dpld^†^	neo-X	CH229-22C23	12,276,220-12,482,724	11	217,193
	neo-Y	CH229-19H21	12,345,041-12,405,202	38	114,562
					
CG3700*	neo-X	CH229-IJ13	17,420,039-17,614,947	21	209,018
	neo-Y	CH229-8B21	17,417,664-17,531,578	33	224,454

*Sanger technology. ^†^454 sequencing. ^‡^Recombinant BAC clone or large deletion on neo-Y.

**Figure 1:**
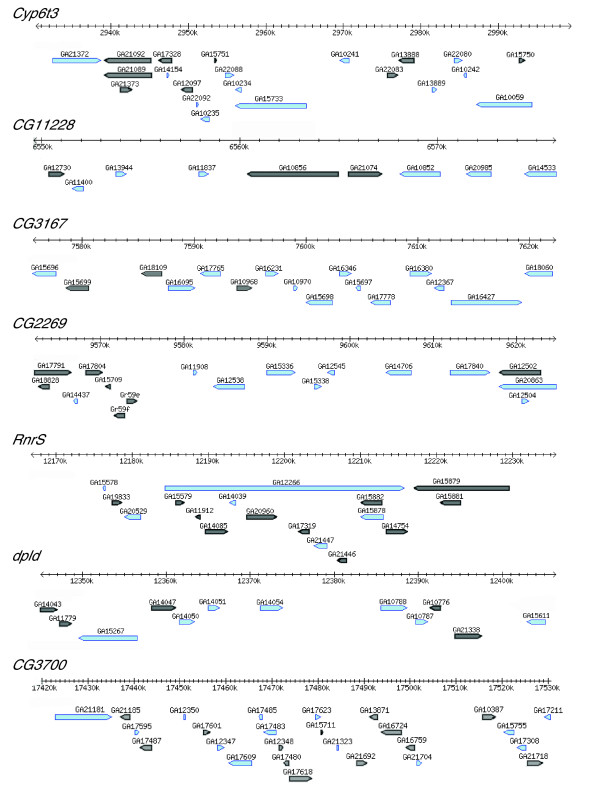
Schematic overview of the genomic regions investigated on the neo-sex chromosomes, anchored on the *D. pseudoobscura *genomic annotation. Genes in grey represent pseudogenes on the neo-Y.

Contigs were assembled and orientated against the homologous *D. pseudoobscura *region using BLAST analysis. Concatenated neo-X and neo-Y contigs were aligned to *D. pseudoobscura*, and annotated using the *D. pseudoobscura *genome annotation as a guide (release r2.0). Figure 1 shows a schematic overview of the orthologous genomic regions investigated on the neo-sex chromosomes, anchored to the *D. pseudoobscura *genome annotation. One BAC clone on the neo-Y, CH229-47N8, spans a 350 kb region in *D. pseudoobscura*, with the middle 300 kb being deleted from the neo-Y. This could reflect a real structural rearrangement on the neo-Y (deletion, duplication or inversion), or instead be an artifact of a recombinant BAC clone.

### Accumulation of transposable elements

Accumulation of repetitive DNA and transposable elements is predicted to occur on a non-recombining genome, such as the Y chromosome. RepeatMasker [16] was used to characterize the distribution of known transposable elements on the neo-sex chromosomes of *D. miranda *(Table 2). As expected for euchromatic regions in Drosophila, transposable element insertions are rare on the neo-X; only about 1% of the DNA analyzed was classified as being transposable element derived. In contrast, about 20% of the DNA derived from the neo-Y chromosome consists of transposable elements, ranging from 14% to 29% among the BAC clones studied (Table 2). There is no significant heterogeneity in the amount of repetitive DNA identified among neo-Y linked regions (χ^2 ^= 8.1; *p *= 0.71) [16].

**Table 2:** Transposable element content in neo-X and neo-Y chromosome contigs

Genomic region*	Retroelements		DNA transposons		% of total sequence
			
	Number	Length (bp)	Number	Length (bp)	
**neo-X linked**					
Cyp6t3	7	931	1	252	0.62
CG11228	12	6,153	0	0	2.88
CG3167	5	722	2	291	0.51
CG2269	12	1,114	1	372	0.68
RnrS	12	2,676	1	74	1.25
dpld	8	1,547	0	0	0.71
CG3700	19	2,852	0	0	1.36
Total	75	15,995	5	989	1.16
					
**neo-Y linked**					
Cyp6t3	51	27,272	2	166	17.94
CG11228	49	19,213	2	125	13.59
CG3167	43	47,660	4	1,068	29.12
CG2269	25	23,855	3	555	15.81
RnrS	50	27,792	5	1,015	17.35
dpld	59	28,969	10	2,337	21.76
CG3700	65	41,649	4	12,119	23.79
Total	342	216,410	30	17,385	20.28

*Genomic regions are named after probes used to isolate BAC clones.

The true total amount of repetitive DNA on the neo-Y, however, is clearly much larger than 20%. In particular, TEs were identified using mainly a *D. melanogaster *transposable element library. Comparisons of the sequenced neo-sex linked regions with the *D. pseudoobscura *genome sequence suggest that up to 50% of the neo-Y chromosome might be made of repetitive DNA. Specifically, the 1.46 Mb of neo-X derived sequence span a 1.40 Mb region in *D. pseudoobscura *(Table 1), suggesting that the neo-X chromosome has a similar repeat content to the autosomal orthologous regions in *D. pseudoobscura*. In contrast, the 0.94 Mb of neo-Y derived sequence span only a 0.42 Mb region in *D. pseudoobscura *(excluding BAC clone CH229-47N8, which either is a recombinant BAC clone or has large genomic deletions on the neo-Y; Table 1). This suggests that more than half of neo-Y derived DNA consists of repetitive and junk DNA, including transposable elements and tandem duplications. Retroelements are the most abundant type of repetitive DNA identified on the neo-Y, accounting for more than 90% of the transposable element DNA of the neo-Y. This accumulation of transposable elements on the neo-Y chromosome of *D. miranda *is consistent with previous observations based on in situ hybridization experiments [8,14] or DNA sequence analysis [8,10].

Several other evolving Y chromosome systems have now been characterized in both plants (*Silene latifolia*, liverwort, papaya [17-19]) and animals (medaka, threespine stickleback [20,21]), and show a similar accumulation of transposable elements. These data suggest that transposable elements are an early invader of newly formed Y chromosomes, and play a general and widespread role in Y chromosome degeneration.

### Degradation of protein-coding genes on the neo-Y

A total of 118 gene pairs were contained within the overlapping regions of the neo-sex linked BAC clones. Of these, 63 are potentially functional on the neo-Y chromosome (that is, they can be translated into a functional protein), while 55 genes are clearly non-functional. In particular, 4 genes are completely deleted from the neo-Y, 27 genes contain frameshift mutations, 7 genes harbor premature stop codons and 17 genes have both frameshift mutations and premature stop codons (Table 3). Thus, almost half of the genes have become pseudogenized after only about one million years of evolution on this non-recombining Y chromosome. This estimate is higher than previous ones (that is, a third of all genes were found to be non-functional [10]), and likely reflects that most previous genes investigated were isolated by PCR, which biases the amplification towards conserved genes.

**Table 3:** Gene count and rates of protein evolution (dN/dS) in the BAC clones analyzed

			Mean (median) dN/dS	
			
Genomic region^a^	No. of genes total	No. of non-functional neo-Y genes	neo-X	neo-Y
Cyp6t3	21	9 (0.43)	0.28 (0.18)	0.63 (0.38)
CG11228	9	3 (0.33)	0.17 (0.14)	0.55 (0.58)
CG3167	16	3 (0.19)	0.12 (0.09)	0.47 (0.36)
CG2269	17	8 (0.47)	0.19 (0.10)	0.39 (0.37)
RnrS	17	11 (0.65)	0.18 (0.05)	0.73 (0.59)
dpld	12	5 (0.42)	0.22 (0.18)	0.47 (0.32)
CG3700	26	16 (0.62)	0.43 (0.15)	0.46 (0.38)
Total	118	55 (0.47)	0.25 (0.14)	0.53 (0.40)

^a^Genomic regions are named after probes used to isolate BAC clones

Combining this new data set with 73 previously studied [10,11,22] and 18 novel genes or gene fragments isolated by PCR (see Materials and methods), the total number of gene pairs investigated to date on the neo-sex chromosomes of *D. miranda *amounts to 209 (that is, roughly 10% of all the genes that were originally present on the neo-Y chromosome). Of these, 123 have a potentially functional copy on the neo-Y chromosome, while 86 have clearly become pseudogenes. The rate of amino acid evolution is significantly higher at non-functional genes on the neo-Y compared to potentially functional ones, with non-functional genes evolving about twice as fast (dN/dS = 0.67 versus dN/dS = 0.38 for non-functional versus functional genes, Wilcoxon test, *p *< 0.001; Table 4). This is consistent with the notion that non-functional genes evolve under little or no selective constraint and thus accumulate amino acid changes in a neutral manner. However, both functional and non-functional genes evolve significantly faster at the protein level on the neo-Y compared to their neo-X homologs (Table 4). Amino acid substitutions accumulate on the neo-Y approximately 2-fold faster than on the neo-X (dN/dS = 0.24 versus dN/dS = 0.50 for neo-X versus neo-Y genes, Wilcoxon test, *p *< 0.001; Table 4). This supports the idea that the efficacy of natural selection is reduced on a non-recombining genome, resulting in an increased rate of accumulation of slightly deleterious amino acid substitutions on the neo-Y. In principal, amino acid accumulation can also result from adaptive protein evolution, and positive selection using polymorphism data has been detected on the neo-Y of *D. miranda *[15]. However, it is likely that this adaptation occurs only at few genes, and the majority of amino acid accumulation is due to the reduced efficacy of natural selection due to selection at linked sites. Thus, most genes in fact suffer from the adaptation that occurs at a few loci on the neo-Y.

**Table 4:** Rate of evolution of functional and non-functional genes on the neo-sex chromosomes

		Mean (median)			Mean (median)		
			
	No. of genes	dS neo-X	dN neo-X	dN/dS neo-X	dS neo-Y	dN neo-Y	dN/dS neo-Y
All	209	1.54 (1.22)	0.31 (0.14)	0.24 (0.10)	2.18 (1.83)	0.81 (0.69)	0.50 (0.37)
Functional	122	1.51 (1.22)	0.22 (0.12)	0.19 (0.10)	2.19 (1.89)	0.61 (0.55)	0.38 (0.25)
Non-functional	87	1.58 (1.27)	0.44 (0.20)	0.31 (0.14)	2.17 (1.75)	1.11 (0.90)	0.67 (0.51)

Interestingly, genes that have a functional copy on the neo-Y chromosome also evolve slower on the neo-X (average dN/dS = 0.19 for functional genes and dN/dS = 0.31 for genes whose homolog is a pseudogene on the neo-Y, Wilcoxon test, *p *< 0.03; Table 4). This suggests that genes that generally evolve under stronger functional constraints (that is, lower dN/dS on the neo-X) are more likely to be conserved on an evolving Y chromosome. This is expected under evolutionary models of Y chromosome degeneration if genes that have fewer amino acid changes on the neo-X are genes in which mutations are more strongly deleterious, since weakly deleterious mutations are more likely to accumulate on a non-recombining chromosome.

Of the 209 genes investigated to date, 118 genes were isolated using the BAC library screen, and 91 genes using mainly a PCR-based approach. A larger fraction of genes is non-functional (47%) isolated from the BAC library, relative to the PCR biased set (35%), although this difference is not statistically significant (*p *= 0.17, Fisher's exact test). This is probably due to both the preferential amplification of conserved genes by PCR, and the fact that for many genes isolated by PCR only partial coding sequence was studied, which means that some stop codons or frameshift mutations will have gone undetected. Also, genes isolated by the BAC library screen evolve faster relative to the PCR biased set (average dN/dS = 0.53 for BAC genes and dN/dS = 0.45 for genes isolated by PCR, Wilcoxon test, *p *= 0.09). Again, a bias towards isolating conserved genes by PCR and a higher fraction of pseudogenes (which have higher dN/dS ratios (see above)) can account for faster rates of protein evolution in the set of genes isolated by the BAC library screen.

### Transposable elements versus Y degeneration

TEs can have both direct and indirect roles in contributing to Y chromosome degeneration. Direct effects are apparent as insertions into coding and regulatory regions on the neo-Y. Using Repeatmasker, we identified 13 TE insertions inside genes on the neo-Y chromosome (all inside introns). The fraction of transposable element insertions is similar between functional and non-functional genes; 7 out of the 51 pseudogenes annotated on the neo-Y contain a TE insertion, and 6 of the 63 genes with intact open reading frames (Fisher's exact test, *p *= 0.3). Thus, transposable elements do not simply accumulate neutrally in inactive genes, but may actively contribute to gene inactivation of adjacent genes on the neo-Y. Transposable elements inserted into pseudogenes are significantly longer than those inserted into functional genes (average length 604 versus 104 bp, Wilcoxon test, *p *= 0.01). We cannot distinguish whether larger TE insertions are simply more tolerated in inactive neo-Y genes, or whether large TE insertions trigger the inactivation of adjacent genes. Gene expression studies should help to clarify this question. TEs can also indirectly interfere with regular gene expression by producing antisense transcripts of adjacent genes, or by altering the chromatin structure of the Y chromosome. We detect no association between the functional status of a gene, and its mean distance to the closest TE insertion. Also, we find no correlation between transposable element abundance and the fraction of pseudogenes or rates of protein evolution of protein-coding genes among the BAC clones investigated. This does not support the idea that an accumulation of TEs indirectly interferes with the function of adjacent genes but, instead, TE insertions might have only very local effects [23]. Detailed gene expression analysis should reveal the effect of individual TE insertions on the function of neo-Y-linked genes.

### Gene Ontology category versus Y degeneration

The contributions of the individual alleles to the phenotype are often non-additive; at many loci, wild-type alleles are dominant and mutant alleles are recessive. The dominance of wild-type alleles has been suggested to be a by-product of the 'physiology of the organism' [24], and that, in dosage-sensitive genes, wild-type alleles should be recessive [24]. Compatible with the physiological theory of dominance, human enzymes are more likely to be haplosufficient (that is, the wild-type is dominant) while structural proteins are more likely to be haploinsufficient, that is, the wild-type is recessive [25]. Genes on the neo-Y chromosome are usually sheltered by a functional copy on the neo-X. Thus, dosage-insensitive genes (like metabolic enzymes) might be more prone to degeneration than dosage-sensitive ones (like regulatory genes). To investigate this prediction, we compared the functional status of a neo-Y gene (that is, functional versus pseudogene) and its rate of amino acid evolution (dN/dS) to the Gene Ontology (GO) annotations. Of the 209 genes investigated, 142 had GO level 2 information. We investigated the same major functional categories as ref. [25]. We observe no significant differences in the proportion of functional and non-functional genes among different functional categories (Table 5). GO categories also do not predict rates of protein evolution of neo-Y genes (results not shown). Thus, there is no evidence in our data that genes that encode proteins with enzymatic functions are more likely to degenerate, or genes encoding regulatory or structural proteins are less likely to degenerate, as might be expected under the physiological theory of dominance [24].

**Table 5:** Major functional categories in functional and non-functional neo-Y linked genes

	Number of genes (percent)		
		
Category	Functional	Non-functional	*P*-value
Binding GO:0005488	53 (60.2)	33 (61.1)	0.90
Catalytic activity GO:0003824	40 (45.5)	24 (44.4)	0.97
Enzyme regulator activity GO:0030234	3 (3.4)	4 (7.4)	-
Molecular transducer activity GO:0060089	14 (15.9)	6 (11.1)	0.48
Motor activity GO:0003774	2 (2.3)	2 (3.7)	-
Structural molecule activity GO:0005198	12 (13.6)	7 (13.0)	0.94
Transcription regulator activity GO:0030528	16 (18.2)	9 (16.7)	0.86
Translation regulator activity GO:0045182	2 (2.3)	1 (1.9)	-
Transporter activity GO:0005215	10 (11.4)	6 (11.1)	0.99

### Sex-biased expression versus Y degeneration

A large fraction of the *Drosophila *genome shows sex-biased expression [26,27]. Since the neo-Y chromosome of *D. miranda *is passed through males only, there may be less selective pressure to maintain genes with female-biased expression on this chromosome. Genes with male-biased expression, in contrast, may be more conserved on the neo-Y than genes with female-biased or unbiased expression [28]. To investigate this prediction, we compared the functional status of a neo-Y gene (that is, functional versus pseudogene) and its rate of amino acid evolution (dN/dS) to patterns of sex-biased gene expression. We classified the neo-sex genes as male-biased, female-biased or non-biased, as identified in gene expression studies in *D. pseudoobscura *[29]. Of the 209 genes investigated, 196 genes had information on patterns of sex-biased gene expression, with 25 genes showing male-biased expression, 52 genes showing female-biased expression, and 119 genes showing no sex bias in patterns of gene expression (Table 6). While genes with female-biased expression show a trend to evolve slightly faster on average on the neo-Y than male-biased or unbiased genes (mean dN/dS = 0.52 versus 0.47 and 0.47; Table 6), these differences in rates of protein evolution are not significant. Also, the fraction of functional genes does not differ significantly among genes with different types of sex-biased expression (Table 6). Thus, there is no evidence in our data set that sex-biased gene expression is a major determinant in predicting patterns of Y chromosome degeneration.

**Table 6:** Rate of evolution of genes with male-, female- and non-biased expression on the neo-Y chromosome of *D. miranda*

			Mean (median)		
			
	Number of genes	Functional (%)	dS neo-Y	dN neo-Y	dN/dS neo-Y
Male-biased	25	60.0	2.25 (1.56)	0.83 (0.67)	0.47 (0.31)
Female-biased	52	61.5	1.85 (1.53)	0.82 (0.64)	0.52 (0.39)
Non-biased	119	58.0	2.28 (2.03)	0.76 (0.71)	0.47 (0.33)

## Conclusion

While extensive sequence analysis has been performed on old Y chromosomes [5,30,31], our data represent the most extensive sequencing effort on a young Y chromosome to date. Several hallmarks of early Y chromosome evolution were identified. At least half of the neo-Y chromosome consists of repetitive DNA, and TEs, in particular, show a striking accumulation on the neo-Y compared to homologous neo-X regions. TEs accumulate uniformly among genomic regions, and no interaction between TE abundance and degeneration at protein-coding genes was detected. Roughly half of all genes originally present on the neo-Y chromosome have already become pseudogenized, after about one million years of evolution on this non-recombining chromosome. Pseudogenes evolve faster at the protein level on the neo-Y compared to functional genes, and the rate of protein evolution at both functional and non-functional genes is significantly elevated on the neo-Y genes relative to their neo-X homologs. This supports theoretical predictions that the efficacy of natural selection is reduced on a non-recombining genome. Genes that are more conserved at the protein level on the neo-X are also more likely to be functional on the neo-Y. However, functional categories or sex-biased gene expression patterns do not predict the amount of degeneration of neo-Y linked genes. It will be of great interest to study patterns of gene expression at these neo-sex linked regions, to link changes at the DNA level to Y inactivation and the evolution of dosage compensation.

How general will our observations on the processes involved in Y degeneration be? One peculiarity of the neo-sex chromosomes in *Drosophila *is that an entire chromosome stops recombining instantaneously, while in other systems, like humans, recombination is restricted gradually. While there is no obvious reason to expect that the types of changes accumulating on the Y would differ between these scenarios, the rate of degeneration is expected to be much slower in systems where only few genes are sheltered from recombination from the beginning [32]. It will be of interest to study such systems, such as, for example, the young sex chromosomes of some plants [33] or vertebrates [20,21].

## Materials and methods

### BAC library construction and screening

The CHORI-229 *D. miranda *BAC library has been constructed from adult males from a *D. miranda *isofemale line (MSH22) by the Children's Hospital Oakland Research Institute. High molecular weight DNA was partially digested with a combination of EcoRI restriction enzyme and EcoRI methylase and size fractionated by pulsed-field electrophoresis. DNA fragments from the appropriate size fraction were cloned into the pTARBAC2.1 vector between the two EcoRI sites. The ligation products were transformed into DH10B (T1 resistant) electro-competent cells (Invitrogen, Carlsbad, CA, USA). High density filters from the BAC library were screened with radiolabeled overgo probes designed to 20 neo-sex linked genes described in ref. [10]. Positive BACs were endsequenced using the T7 and SP6 primers, and the sequences were blasted against the *D. pseudoobscura *genome sequence to orient the BAC clones along chromosome 3 of *D. pseudoobscura *(which is homologous to the neo-sex chromosomes of *D. miranda*). BAC clones were typed as neo-X or neo-Y specific using sex-specific polymorphisms. A total of 202 BAC clones were mapped and oriented to the neo-sex chromosomes of *D. miranda*.

### BAC sequencing

Seven neo-X and seven neo-Y BACs were chosen for sequencing, employing two different sequencing strategies. Three homologous BAC clone pairs were sequenced using Sanger technology (clones isolated with probes *CG3700*, *RnrS*, and *Cyp6t3*). Purified BAC DNA was randomly sheared using hydroshear technology, size selected (approximately 2.5 kb) and subcloned into a pUC based vector and transformed into *Escherichia coli*. Universal primers and BigDye Terminator chemistry (Applied Biosystems, Foster City, CA, USA) were used for sequencing randomly selected plasmid subclones to an average sequence depth of 6×. The Phred/Phrap/Consed suite of programs was used for assembling and editing the sequences [34]. Four homologous BAC clone pairs were sequenced using 454 technology (clones isolated with probes *CG11228*, *CG2269*, *dpld*, *CG3167*). 454 Life Sciences (Branford, CT, USA) performed one large (70 × 75) run of the BAC DNA, partitioned into eight. A total of 292,705 quality filtered sequence reads were generated with an average length of approximately 102 bp. The total sequence output was 30,171,327 bases. After *E. coli *removal, all quality filtered sequences were assembled using the 454 Newbler assembler. Sequences have been deposited in GenBank under accession numbers EU624504 - EU624734.

### Repeat analysis, assembly, and global alignment

To identify repetitive elements, the neo-X and neo-Y sequence contigs were analyzed with RepeatMasker [16] using the *Drosophila *library. The masked BAC sequence contigs were oriented and mapped against the *D. pseudoobscura *genome sequence using BLAST analysis. Contigs with significant BLAST matches were concatenated to generate single sequence contigs for each BAC region from the neo-X and neo-Y chromosomes. A global alignment of the final neo-X, neo-Y and *D. pseudoobscura *sequence was performed using PipMaker [35].

### Annotation and coding sequence analysis

The neo-sex linked BAC clones were annotated using the *D. pseudoobscura *genome sequence annotation as a guide. Pairwise alignments between *D. pseudoobscura *and the neo-X or neo-Y sequence, respectively, were performed using PipMaker. The positions of exon-intron boundaries on the neo-X and neo-Y sequence were determined using the alignment, and coding regions were extracted using custom PERL scripts. Coding regions were realigned using MUSCLE and by eye. Genes that contain *STOP *codons or frameshift insertions or deletions on the neo-Y were classified as non-functional. To estimate rates of synonymous and nonsynonymous mutations (*dS *and *dN*) on the neo-X and neo-Y branch, we used the *PAML *software package [36], which accounts for unequal transition and transversion rates and unequal base and codon frequencies.

### Gene expression and GO analysis

To classify genes as female-, male- or non-biased, we used microarray data from *D. pseudoobscura *[29]. GO classes were extracted from FlyBase [37].

## Abbreviations

BAC, bacterial artificial chromosome; GO, Gene Ontology; TE, transposable element.

## Authors' contributions

DB conceived and supervised the study. XM isolated genomic DNA from *D. miranda *for BAC library construction. PJ made the *D. miranda *BAC library and performed the BAC library screening. EH isolated BAC clone DNA. DB analyzed the data, and KMW helped with the analysis of the GO data. DB wrote the manuscript.
